# IMPLEMENTATION BOOSTER: TESTING A VIRTUAL SIMULATION GAME TO ENHANCE CONFIDENCE AND COMPETENCE

**DOI:** 10.1093/geroni/igae098.0478

**Published:** 2024-12-31

**Authors:** Grace Armstrong, Mary McCormack, Ilona Seaman, Mary Dolansky, Anne Pohnert, Nicholas Schiltz, Sarah Ball

**Affiliations:** Case Western Reserve University, Cleveland, Ohio, United States; CVS Health Minute Clinic, Woonsocket, Rhode Island, United States; Case Western Reserve University, Cleveland, Ohio, United States; Case Western Reserve University, Cleveland, Ohio, United States; CVSHealth, Bellingham, Massachusetts, United States; Case Western Reserve University, Cleveland, Ohio, United States; CVSHealth Minute Clinic, Woonsocket, Rhode Island, United States

## Abstract

The Virtual Clinic, a serious video game, aimed to improve provider competency and confidence in the delivery of AFHS 4Ms care through simulation of realistic older adult patient visits. The Virtual Clinic (VC) was developed in response to focus groups of front-line providers that revealed they valued gaining competency in delivering 4Ms through practice. The first prototype was tested in July 2021. Based on feedback the prototype was iteratively modified, tested and evaluated for effectiveness over three additional pilot waves. Regions of low 4Ms delivery were specifically targeted in an open-enrollment style of recruitment, with various incentives depending on the wave. A total of 262 providers have participated. Providers’ performance were evaluated one week before, one week after, and four weeks after participation in the virtual clinic. Participating providers were matched at a 3:1 ratio to providers in the system that did not participate in the VC and had similar baseline 4Ms delivery and patient volume. Across all waves, participants in the VC were found on average to have a 24% increase in M-score, and a 7% increase in eligible appointments with at least one M delivered four weeks after participation in the virtual clinic. Overall, participation in the virtual clinic shows moderate, but statistically significant increase in the delivery of evidence-based 4Ms care in providers with low baseline performance.

